# Barriers Over Time to Full Implementation of Health Information Exchange in the United States

**DOI:** 10.2196/medinform.3625

**Published:** 2014-09-30

**Authors:** Clemens Scott Kruse, Verna Regier, Kurt T Rheinboldt

**Affiliations:** ^1^School of Health AdministrationCollege of Allied Health ProfessionsTexas State UniversitySan Marcos, TXUnited States

**Keywords:** medical informatics, electronic health record (EHR), electronic medical records (EMR), health information technology (HIT), quality improvement, national health policy, workflow, past trends

## Abstract

**Background:**

Although health information exchanges (HIE) have existed since their introduction by President Bush in his 2004 State of the Union Address, and despite monetary incentives earmarked in 2009 by the health information technology for economic and clinical health (HITECH) Act, adoption of HIE has been sparse in the United States. Research has been conducted to explore the concept of HIE and its benefit to patients, but viable business plans for their existence are rare, and so far, no research has been conducted on the dynamic nature of barriers over time.

**Objective:**

The aim of this study is to map the barriers mentioned in the literature to illustrate the effect, if any, of barriers discussed with respect to the HITECH Act from 2009 to the early months of 2014.

**Methods:**

We conducted a systematic literature review from CINAHL, PubMed, and Google Scholar. The search criteria primarily focused on studies. Each article was read by at least two of the authors, and a final set was established for evaluation (n=28).

**Results:**

The 28 articles identified 16 barriers. Cost and efficiency/workflow were identified 15% and 13% of all instances of barriers mentioned in literature, respectively. The years 2010 and 2011 were the most plentiful years when barriers were discussed, with 75% and 69% of all barriers listed, respectively.

**Conclusions:**

The frequency of barriers mentioned in literature demonstrates the mindfulness of users, developers, and both local and national government. The broad conclusion is that public policy masks the effects of some barriers, while revealing others. However, a deleterious effect can be inferred when the public funds are exhausted. Public policy will need to lever incentives to overcome many of the barriers such as cost and impediments to competition. Process improvement managers need to optimize the efficiency of current practices at the point of care. Developers will need to work with users to ensure tools that use HIE resources work into existing workflows.

## Introduction

Health Information Exchange (HIE) is not a new concept. It was prioritized in a national agenda in the United States by President Bush in 2004 [1]. Physicians understand and agree with the altruistic benefit that HIE can enable [2], but many barriers prevent its widespread adoption. Enterprise-wide savings have full implementation range of $8.1-$77.8 billion [3,4] and a pay-back period as low as 2.1 years [5], but the disjointed nature of the health system in the United States creates a disconnect between long-term savings of payers and short-term investment of providers. Many studies have examined the barriers to adoption, but no research has examined these barriers over time.

An HIE is the electronic transfer of clinical and administrative information [3], across diverse and often competing health care organizations [2], at the state or regional levels [6], delivering the right information to the right person at the right time. The use of HIE networks has the potential to reduce up to 18% of patient safety errors generally and as many as 70% of preventable adverse drug events across the care continuum [7]. The HIE concept has the potential to reduce health care costs in the United States through a reduction in unnecessary medical tests and procedures, by improving communication about patients’ latest medication regimens, laboratory test results, and diagnostic procedures [7]. The HIE concept also has the potential to improve infection control practice. For example, Kho et al found that across a large metropolitan area, 286 unique patients generated 587 admissions accounting for 4335 inpatient days where the receiving hospital was not aware of the prior history of methicillin-resistant Staphylococcus aureus (MRSA) [8].

The Institute of Medicine (IOM) determined that automation of clinical data through electronic methods would result in better patient care [8]. What followed in 2004, was Executive Order 13335 which set a goal to fully adopt electronic health records (EHR) within ten years [1]. Within a short amount of time, several HIEs, under both public and private funding, appeared on the health care landscape in the United States. There was no standard for an organization that enabled the exchange, or the exchange itself. Lack of standards continues today, which enables innovation in design, but also does not help new startup initiatives start with a successful model. Studies demonstrated the advantages to the concept of health information exchange: cost, quality, safety, better patient care, fewer repeat tests, reduced readmissions, and the ability to identify “drug hoppers” [1-5]. The HIE is defined in concept, as in the previous paragraph, but not in design.

In the first few years after President Bush’s Executive Order, most HIEs initiated had failed due to their own fiscal weight and the absence of a viable business plan. Barriers to adoption were listed in the literature: lack of a viable business plan to sustain the HIE and acceptance by providers and patients [6], privacy/security concerns [8,9], usability [10], lack of technical support or technology gaps [9,10], missing data [11,12], disruption of workflow [9,10,12], startup costs from public and private dollars [13,14], lack of experience in the concept of HIE, and interference with competition [14].

In 2009 the United States Congress passed the Health Information Technology for Economic and Clinical Health (HITECH) Act, as part of the American Recovery and Reinvestment Act (ARRA), which earmarked $19.2 billion as incentives for providers to adopt the EHR and to participate in HIE [1]. The National Coordinator for Health Information Technology (ONC) created the State HIE Cooperative Agreement Program which sponsored public grants specifically for the startup of HIEs and Regional Health Information Organizations (RHIOs). States were encouraged to match the federal dollars to also incentivize the HIE concept. The intent was to help new HIE initiatives overcome the initial fiscal problems until the concept of HIE was accepted and supported in the health care community. The act also enables the comingling of private and public funds because the public money was issued as a grant. Private organizations interested in the pursuit of an exchange could augment their own budget with public grant money to provide an advantageous fiscal position not previously available.

The HITECH Act promotes the electronic exchange of clinical health data across organizations with the expectation that access to comprehensive patient information will help clinical decision-making. Once again, the federal government defined what the HIE should do, but stopped short of defining how it should be done. There is general agreement that access to a patient’s medical record at the point of care will help to avoid duplicative tests, increase administrative efficiency, improve disease management, and ultimately result in cost savings. Interoperable health information may also help to identify and avoid medication complications, thus increasing patient care and safety. However, the fragmented health system in the United States presents many structural, economic, and cultural challenges to achieving a robust environment of electronic data exchange [4].

In light of federal efforts to facilitate the adoption of EHRs and formation of HIEs, the ONC, under the auspices of the Department of Health and Human Services, tracks EHR adoption rates for office-based providers and hospitals on its Health IT Dashboard. In 2008, 17% of office-based providers used a basic EHR, increasing to 40% in 2012. Similarly, in 2008, 13% of hospitals implemented a basic EHR, growing to 56% in 2012. A basic EHR includes patient demographics, patient problem lists, medication histories, clinical notes, electronic orders for prescriptions, laboratory results, and imaging results [15]. In regard to the advancement of HIEs, the 2013 e-Health Initiative Survey on Health Data Exchange identified 84 data exchange initiatives out of 315 that are at advanced stages of operation and thus able to support data exchange. This represents an increase from 57 advanced initiatives in 2011. Growth trends indicate a positive relationship between EHR adoption and HIEs with exchange capacity [16].

However, it is important to note the distinction between HIE capacity to exchange data from the actual rates of data exchange by providers and health organizations. The absence of a viable business plan or standard organizational structure of the exchange may have caused the rate of exchange to be lower than desired. A recent study identified similar growth in hospitals exchange of health information with other entities. Exchange rates to providers outside the hospital’s organization were 41% in 2008 and increased to 58% in 2012. In contrast, data on HIE utilization rates among office-based providers is more limited to narrower studies that focus on specific specialties, user types, and geographic regions [17].

Although research has analyzed the EHR, HIE, and barriers to adoption of both, no study maps the barriers reported over time. This gap in the literature provides the basis for this article. The aim of this study is to examine the frequency of barriers as listed in published material from PubMed (MedLine), CINAHL, and Google Scholar. From this analysis, a data map over time is developed to better understand the dynamic nature of the results. The results of this study enable future researchers to develop empirical models and policy makers to exploit the successful levers that generate a desired result.

## Methods

Search terms were selected based on the experience of the authors in the field of health care administration. The time-frame for the literature review of 1993-2014 was selected out of convenience. It was assumed that two decades would be sufficient to capture trends. The years under study were Jan 2009-Mar 2014. This span was chosen because of the incentives (grants) enabled by the ARRA, and also a concentrated study on these years was expected to enhance the results.

**Figure 1:**
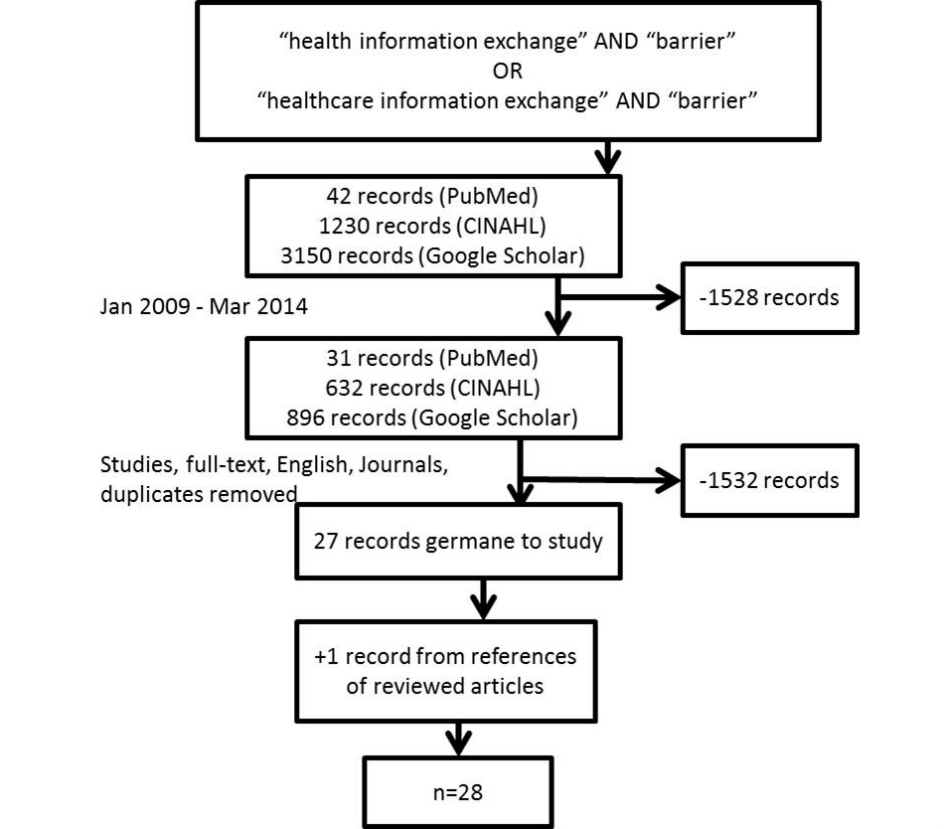
The literature review process.

Figure 1 illustrates the literature review process that identified sources consisting of empirical studies, articles, editorials, commentaries, opinion papers, organizational theories, and text books. The window of time for this study eliminated 1528 records. Focusing on studies, full-text, English, academic journals, and eliminating duplicates resulted in the removal of an additional 1532 records. After 27 articles were identified and reviewed, one additional article was selected from the references of multiple studies. The final sample was 28.

There were no human subjects in this study; all information came from secondary data sources. The studies used in this research were sources that were publically available, and the subjects could not be identified either directly or through identifiers linked to the subject. This qualifies under “exempt” status in 45 CFR 46. Therefore, IRB review was not required, and consent from subjects was irrelevant.

## Results

Table 1 illustrates the results organized by by the year in which the literature was published. This table lists the associated number, synchronized with the references section, the journal publishing the article, the associated method, and the barriers listed. For example, Rudin R et al. identified an inequity between providers of the information and others (in disparate organizations) that benefit from the presence of the information. This was categorized into the barrier of “impedes competition” [12]. Patel V et al identified an inequity of those who pay for participation in the HIE and those who benefit, such as the patient. This was categorized as both “impedes competition” and “misaligned incentives” [13]. Numbers in the column labeled “Art#” are not in order because the article was also used in the literature review, and the authors wanted to synchronize the list of articles in this study with its references section.

The authors categorized the barriers into 16 common themes, and listed in parentheses the barrier interpreted from the studies’ results. A total of 28 articles identified 16 unique barriers. Barriers are listed in the order of most identified. The numbers in each column correspond to the study itself, synchronized with the references. Three articles in Table 1 list the numbers in italics. These articles were literature reviews. The authors chose to include these studies for reasons of consistency and reliability.

The number of articles identified and reviewed from 2009-2014 were 1, 5, 7, 7, 7, and 1, respectively, while the number of barriers listed from 2009-2014 were 2, 12, 11, 8, 7, and 2, respectively. In 2009, the only barriers listed were cost and physician resistance [4]. In 2010, the 12 barriers were: cost, efficiency/workflow, lack of technical support/technology gap, impedes competition, value of HIE is difficult to measure, privacy/security, usability, heavily dependent on leadership of the organization, liability, physician resistance, decreases quality, and increases error [2,10,18-20]. In 2011, the 11 barriers listed were: cost, efficiency/workflow, lack of technical support/technology gap, impedes competition, value of HIE is difficult to measure, privacy/security, clinical data missing when needed, usability, heavily dependent on leadership of the organization, liability, misaligned incentives [12,13,21-25]. In 2012, the 8 barriers listed were: cost, efficiency/workflow, lack of technical support/technology gap, impedes competition, value of HIE is difficult to measure, privacy/security, clinical data missing when needed, and liability [14,26-31]. In 2013, the barriers listed were cost, efficiency/workflow, impedes competition, value of HIE is difficult to measure, clinical data missing when needed, usability, heavily dependent on leadership of the organization, lack of standards, and misaligned incentives [32-38]. In 2014, the two barriers listed were efficiency/workflow and usability [39]. Table 2 organizes the barriers listed in the literature by the year in which the literature was published.

**Table 1:** Studies and barriers identified.

Art #	Study	Date	Barriers
4	Adler-Milstein J, Bates DW, Jha AK. Regional Health Information Organizations: progress and challenges	2009	viable business model, failure to obtain sufficient participation, cost
2	Fontaine P, Ross SE, Zink T, Schilling LM. Systematic review of health information exchange in primary care practices	2010	cost, security and privacy issues, liability, leadership, strategic planning, and competition, technical gap
7	Vest J, Gamm L. More than just a question of technology: Factors related to hospitals’ adoption and implementation of health information exchange	2010	cost, competition, privacy concerns, legal liability
10	Ross SE, Schilling LM, Fernald DH, Davidson AJ, West DR. Health information exchange in small-to-medium sized family medicine practices: motivators, barriers, and potential facilitators of adoption	2010	cost, workflow, tech support, competition, non-solidarity, usability
11	Tham E, Ross SE, Mellis BK, Beaty BL, Schilling LM, Davidson AJ. Interest in health information exchange in ambulatory care: a statewide survey	2010	missing data
18	Wright A, Soran C, Jenter C. Physician attitudes toward health information exchange: results of a statewide survey	2010	privacy, difficulty to assess value of HIE
19	Dixon B, Zafar J. A framework for evaluating the costs, effort, and value of nationwide health information exchange	2010	technology gap
12	Rudin R, Volk L, Simon S, Bates D. What affects clinicians’ usage of health information exchange	2011	gaps in data, workflow, usability, billing (cost), inequity between providers of information and those who benefit from the information (competition)
13	Patel V, Abramson EL, Edwards A, Malhotra S, Kaushal R. Physicians’ potential use and preferences related to health information exchange	2011	costs, tech support, inequity of those who pay , and those who benefit (impedes competition and misaligned incentives), workflow, usability
20	Joshi JK. Clinical Value-Add for Health Information Exchange (HIE)	2011	quality of care, effect on patients, cost, error, organizational efficiency, acceptance by physicians and patients.
21	Korst LM, Aydin CE, Signer JM, Fink A. Hospital readiness for health information exchange: Development of metrics associated with successful collaboration for quality improvement	2011	strong leadership, tech support, value of data
22	Adler-Milstein J, Bates DW, Jha AK. A survey of health information exchange organizations in the United States: implications for meaningful use	2011	cost, leadership, lack of value
23	Lluch M. Health care professionals’ organisational barriers to health information technologies-A literature review	2011	structure of health care organizations (ownership), tasks (workflow), people policies (liability), incentives (cost), information and decision processes (tech support)
24	Gadd CS, Ho YX, Cala CM, Blakemore D, Chen Q, Frisse M, Johnson K. User perspectives on the usability of a regional health information exchange	2011	not user-friendly (efficiency), need additional tech support, data incomplete (data missing when needed)
25	Hincapie AL, Warholak TL, Murcko AC, Slack M, Malone DC. Physicians’ opinions of a health information exchange	2011	lack of value, technology gaps, gaps in data
14	Pevnick J, Claver M, Dobalian A, Asch S, Stutman H, Tomines A, Fu P. Provider stakeholders’ perceived benefit from a nascent health information exchange: A qualitative analysis	2012	legal concerns (liability), data security, costs, competition, bureaucracy (efficiency)
26	Williams C, Mostashari F, Mertz K, Hogin E, Atwal P. From the ONC: The strategy for advancing the exchange of health information	2012	tracking source of information (missing data), patient matching (privacy), workflow, liability
27	Steward W, Koester K, Collins A, Myers J. The essential role of reconfiguration capabilities in the implementation of HIV-related health information exchanges	2012	cost, technology gap, value, workflow
28	Deas TM, Solomon MR. Health information exchange: foundation for better care (Perspectives)	2012	cost, difficult to place value on HIE, missing data
29	Kralewski JE, Zink T, Boyle R. Factors Influencing Electronic Clinical Information Exchange in Small Medical Group Practices	2012	cost, lack of value, competition, technology gap, privacy
30	Myers JJ, Koester KA, Chakravarty D, Pearson C, Maiorana A, Shade S, Steward W. Perceptions regarding the ease of use and usefulness of health information exchange systems among medical providers, case managers and nonclinical staff members working in HIV care and community settings	2012	usefulness (value), difficulty of interaction with HIE (tech support), workflow
31	Vest J, Jasperson JS. How are Health Professionals Using Health Information Exchange Systems? Measuring Usage for Evaluation and System Improvement	2012	effectiveness of Master Patient Index (MPI) (privacy), tech support
32	Adler-Milstein J, Bates DW, Jha AK. Operational Health Information Exchanges show substantial growth, but long-term funding remains a concern	2013	cost, provider pays while payer benefits, difficult to measure value
33	Dixon BE, Jones JF, Grannis SJ. Infection preventionists' awareness of and engagement in health information exchange to improve public health surveillance	2013	lack of awareness, decision support (workflow), usability, interoperability (standards), missing data
34	Furukawa MF, Patel V, Charles D, Swain M, Mostashari F. Hospital electronic health information exchange grew substantially in 2008-12	2013	limited interoperability (standards), competition, cost
35	Campion T, Edwards A, Johnson S, Kaushal R. Health information exchange system usage patterns in three communities: practice sites, users, patients, and data	2013	workflow
36	Miller A, Tucker C. Health information exchange, systems size and information silos	2013	standards, competition
37	Ben-Assuli O, Shabtia I, Leshno M. The impact of EHR and HIE on reducing avoidable admissions: controlling main differential diagnosis	2013	costs, missing data, decision making (workflow), leadership, competition
38	Vest JR, Campion TR, Kaushal R. Challenges, Alternatives, and Paths to Sustainability for Health Information Exchange Efforts	2013	cost, lack of value, competition
39	Thorn SA, Carter MA, Bailey JE. Emergency Physicians' Perspectives on Their Use of Health Information Exchange	2014	workflow, usability

**Table 2:** Barriers by the year published.

Barriers			2009	2010		2011			2012	2013			2014	Instances of the barrier	
Cost			4	2, 10, 20		12, 13, 22, 23			27, 28, 29	32, 34, 37, 38				15	15%
Efficiency/workflow				10, 20		13, 23, 24			14, 26, 27, 30	33, 35, 37			39	13	13%
Lack of technical support/tech gap				2, 10, 19		12, 13, 21, 23, 24, 25			27, 29, 30, 31					13	13%
Impedes competition				2, 10		12, 13			14, 28	34, 36, 37, 38				10	10%
Value of HIE is difficult to measure				18		21, 22, 25			27, 28, 29, 30	32, 38				10	10%
Privacy/security concerns				2, 18		23			14, 26, 29, 31					7	7%
Clinical data missing when needed						12, 24, 25			26, 28	33				6	6%
Usability				10		12, 13				33			39	5	5%
Heavily dependent on leadership of the organization				2		21, 22				37				4	4%
Liability concerns				2		23			14, 26					4	4%
Lack of standards										33, 34, 36				3	3%
Physician resistance			4	20										2	2%
Misaligned incentives						13				32				2	2%
Decreases quality				20										1	1%
Increases error				20										1	1%
Lack of awareness										33				1	1%
# barriers (n=16)	2, 13%	12, 75%			11, 69%		8, 50%	10, 63%			2, 13%	97			
# articles (n=28)	1, 4%	5, 18%			7, 25%		7, 25%	7, 25%			1, 4%				

In 2009, only 2 out of 16 barriers (13%) were listed by only 1 of 28 articles (4%). In 2010, 12 of 16 barriers (75%) were listed by 5 out of 28 articles (18%). In 2011, 11 of 16 barriers (69%) were listed by 7 of 28 articles (25%). In 2012, 8 of 16 barriers (50%) were listed by 7 of 28 articles (25%). In 2013, 7 out of 16 barriers (44%) were listed by 7 of 28 articles (25%). In 2014, 2 of 16 barriers (13%) were listed by only 1 of 28 articles (4%).

The barrier of cost was listed in the 2011 literature four times; one of which was a literature review. Cost was listed in the 2013 literature four times, the 2010 and 2012 literature three times, and in 2009 only once; in 2010-2012 one article was a literature review. If literature reviews were removed from the analysis, there would be a general increase in frequency of the barrier cost. From 2009-2014, the barrier “cost” was listed 15 of 97 instances (15%), “efficiency/workflow” and “lack of technology support / technology gap” were listed 13 of 97 instances (13%), “impedes competition,” and “value of HIE is difficult to measure” were identified 10 of 97 instances (10%), “privacy/security issues” was listed 7 of 97 instances (7%), “clinical data missing” was listed 6 of 97 instances (6%), “usability” was listed 5 of 97 instances (5%), “heavily dependent on leadership” and “liability” were listed 4 of 97 instances (4%), “lack of standards” was listed 3 of 97 instances (3%), “physician resistance” and “misaligned incentives” were listed 2 of 97 instances (2%), “decreases quality,” “increases error,” and “lack of awareness” were each listed 1 of 97 instances (1%). The year 2010 revealed the greatest quantity of barriers listed (75%) and the years 2011-2013 tied for the number of articles published with barriers (25%).

## Discussion

The concern of cost is discussed consistently in the literature, mostly with the concern of “no viable business plan” listed as the reason. This is not surprising. Very little participation in HIEs occurred prior to the ARRA in 2009, and most folded due to a lack of funding. The HITECH Act provided seed money, and the federal government asked the states to match or significantly contribute to the establishment of HIEs throughout the country. The stimulus money evaporates in 2014. By the end of 2014, HIEs will either develop a viable business plan or close their activities.

The second most consistent barriers discussed were efficiency/workflow, impedes competition, and value difficult to measure. These barriers were discussed four of the six years analyzed. The concept of participation in the HIE is intended to provide better quality care, but it makes no promises of efficiency. The concern that HIE participation will impede competition is concerning because it flies in the face of the altruistic nature of health care. This factor may be unique in the United States due to the competitive nature of the health care industry, the philosophy of health care as a privilege, and the nonholistic definition of medicine that focuses on the identification and treatment of disease rather than promotion of overall health. Those health care organizations that treat Center for Medicaid and Medicare Services (CMS) beneficiaries submit for reimbursement based on diagnosis, which implies the presence of disease. The difficult nature of measuring the benefit of HIE is also not surprising. The use of HIE resources would not manifest itself in an obvious improvement of care; instead, the use of HIE would most likely result in fewer tests, for those that require tests, and the decrease of drug abuse for those who “shop” for controlled medications by frequent visitation of disparate emergency departments. The cost of participating in the HIE may indeed be more than the cost of duplicating the tests. In this regard, difficulty in measuring the value of the HIE may also point to the issue of cost.

The technical aspect of HIE was also listed frequently. This is an interesting item to be listed as a barrier which should have been addressed by the HITECH Act with the establishment of the Regional Extension Centers (RECs). These RECs provide technical support specifically to organizations transitioning to electronic health records and those interested in participating in an HIE. The frequency of this barrier dropped off after 2011, which could be a result of inter-organizational relationships being established with the operation of the RECs.

Privacy/security concerns is also not surprising. The Health Information Portability and Accountability Act (HIPAA) of 1996 creates an atmosphere of hypersensitivity for patient privacy and the security of health related information. An interesting observation is that the barrier “lack of standards” did not appear in the literature until 2013. This barrier could be accounted for by awareness, but the barrier “lack of awareness” also did not surface until the same year, although with only one third the frequency. Logically, an increase of awareness would result in an increase of standards development which should decrease the concern of privacy/security. The frequency of privacy/security did drop off after 2012, which might be indicative of the latter logic trail.

A limitation of this study is that it analyzes the frequency of barriers based on the year in which the articles were published, but it does not evaluate the year in which data were collected/analyzed. The editorial process varies by journal and quality of the initial draft. This could add 6 months or more to the publication process. A deeper analysis of the data-collection aspect of each article could make the focus of a future study. Another limitation is that the publication process will decay the internal validity of the study; for instance, more material will be published in 2014 during the editing/reviewing process that could contribute to the study.

The internal validity of this study seems otherwise sound. The inclusion of other literature reviews illustrates the exhaustive nature of the barriers mentioned. The only exception is the literature review by Joshi et al [20]. This article was published in 2010, but it could have easily focused on barriers mentioned prior to 2009, which was the earliest inclusion criteria for this study.

The external validity of this literature review seems strong. Studies included in this review included countries external to the United States; however, barriers in these countries might be juxtaposed to those in the United States due to the competitive nature of the health care industry in the United States.

The frequency of the barrier “cost” may identify problems in the future. By the end of 2014, federal funds for HIE initiatives will cease, which could cause the number of HIEs in the United States to plummet due to the lack of viable business plans. If the United States wants to ensure the longevity of HIEs in the country, it may need to lever additional incentives aimed at providers, or require health plans to contribute to the HIE programs. This raises several policy issues for the government to consider. Master patient indices at HIEs will only be effective if common data standards are in place across the nation.

The frequency of the barrier “efficiency/workflow” may be indicative of waste in the process of exchanging clinical data. Process improvement managers, or those familiar with Lean practices, need to map existing processes and take steps to eliminate wasted steps or procedures. By optimizing the process at the point of care, providers can feel confident that existing workflow procedures are efficient. If tools used to access data through HIEs are part of an inefficient process, the tools will simply transform them into expensive inefficient processes.

Additional measures could be taken by developers to alleviate the barrier “efficiency/workflow.” Developers should increase their efforts to collect user needs and established workflows of users. Additional efforts at this step of software development could ensure ease of use for tools that access and contribute to HIE resources. These tools should be integrated into existing workflows, and the tools should be easily navigable. Accessing data through HIE should augment the effectiveness of care, and should not decrease the efficiency of care. The absence of concerns about privacy and security may only indicate the steady state of expectations of the same.

To address the barrier “lack of technical support,” managers at RECs should focus closely on the local HIE efforts and reach out to the corresponding Regional Health Information Organizations (RHIOs). The RECs should help organizations realize improvements in both efficiency and effectiveness through the use of HIEs. The managers at Regional Health Information Organizations (RHIOs) should realize that the services that they provide should not take any longer to access than the repeated tests that the HIEs are supposed to mitigate. Managers at RHIOs should also reach out to senior leadership at organizations that could participate in HIEs to win their confidence. Once senior leadership is convinced of the value of HIE, our nations should see additional participation and inter-organizational trust that would overcome competitive environments.

## Abbreviations

ARRA: American recovery and reinvestment act
CFR: code of federal regulation
CINAHL: cumulative index for nursing and allied health literature
CMS: Center for Medicaid and Medicare services
EHR: electronic health record
EMR: electronic medical record
HC: health care
HIE: health information exchange
HIPAA: health insurance portability and accountability act
HIT: health information technology
HITECH: health information technology for economic and clinical health
MPI: master patient index
IOM: institute of medicine
IRB: institutional review board
MRSA: Methicillin-resistant Staphylococcus aureus
ONC: office of the national coordinator
REC: regional extension center
RHIO: regional health information organization
